# *Scandinavian Journal of Trauma, Resuscitation and Emergency Medicine* reviewer acknowledgement 2015

**DOI:** 10.1186/s13049-016-0223-6

**Published:** 2016-03-17

**Authors:** Kristi G. Bache

**Affiliations:** Norwegian Air Ambulance Foundation, Drøbak, Norway

## Abstract

The editors of *Scandinavian Journal of Trauma, Resuscitation and Emergency Medicine* would like to thank all our reviewers who have contributed to the journal in volume 23 (2015).

**Muhammad Adrish**

United States of America

**Pål Ager-Wick**

Norway

**Juan Asensio**

United States of America

**Pongsakorn Atiksawedparit**

Thailand

**Graham Barker**

United Kingdom

**Joost Bierens**

Netherlands

**Paolo Boffano**

Italy

**Katarina Bohm**

Sweden

**Mikkel Brabrand**

Denmark

**Kolbjørn S Brønnick**

Norway

**Andrzej Cacko**

Poland

**Muazez Cevik**

Turkey

**Kirsty Challen**

United Kingdom

**Nipon Chattipakorn**

Thailand

**Jitender Chaturvedi**

India

**Ivy Cheng**

Canada

**Damian Clarke**

South Africa

**Tim Coats**

United Kingdom

**Maria J Colomina**

Spain

**Luiz Guilherme Costa**

Brazil

**Charles Court-Brown**

United Kingdom

**David Dries**

United States of America

**Francois-Xavier Duchateau**

France

**Martin Dworschak**

Austria

**Evert Eriksson**

United States of America

**Espen Fevang**

Norway

**Gary Ford**

United Kingdom

**Spiros Frangos**

Afghanistan

**Margaret Fry**

Australia

**Samuel Galvagno**

United States of America

**Alexandre Ghuysen**

Belgium

**George Giannopoulos**

Greece

**David Griffiths**

United Kingdom

**Carl Gwinnutt**

United Kingdom

**Samir H Haddad**

Saudi Arabia

**Kristiann Heesch**

Australia

**Johan Herlitz**

Sweden

**Lars Jacobsen**

Norway

**Hwan Jun Jae**

Korea, South

**Marcin Jaracz**

Poland

**Iosifina Karmaniolou**

Greece

**Jeffry Kashuk**

United States of America

**Marius Keel**

Switzerland

**Kyuseok Kim**

Korea, Republic Of

**Firas Kobeissy**

Lebanon

**Antti Koivusalo**

Finland

**Rudolph Koster**

Netherlands

**Jinn-Rung Kuo**

Taiwan

**Jouni Kurola**

Finland

**Lionel Lamhaut**

France

**Mark Lehnert**

Germany

**Ari Leppaniemi**

Finland

**Chun Lim**

United States of America

**Gitte Linderoth**

Denmark

**Anne Lippert**

Denmark

**Andrew Lockey**

United Kingdom

**Sigbjørn Løes**

Norway

**Trine Madsen**

Denmark

**Ronald Maier**

United States of America

**Ajai Malhotra**

United States of America

**Aidan Marsh**

United Kingdom

**Tara McMorrow**

Ireland

**Carl Mcqueen**

United Kingdom

**Jane McVicar**

United Kingdom

**Terje Meling**

Norway

**Bruno Meloni**

Australia

**Pavel Michalek**

Czech Republic

**Timothy Miller**

United States of America

**Biswadev Mitra**

Australia

**Patrick Murphy**

United Kingdom

**Yih Yng NG**

Singapore

**Alexander Olaussen**

Australia

**Peter Paal**

Austria

**Cameron Palmer**

Australia

**Michael Pasquale**

United States of America

**Tommaso Pellis**

Italy

**Thomas Rea**

United States of America

**Kristina Rehm**

United States of America

**Marius Rehn**

Norway

**Giuseppe Ristagno**

Italy

**Lindsetmo Rolv-Ole**

United States of America

**Kurt Ruetzler**

United States Minor Outlying Islands

**John Santamaria**

Australia

**Danielle Sartorius**

Switzerland

**Thomas Scalea**

United States of America

**Thomas Andersen Schmidt**

Denmark

**Herbert Schöchl**

Austria

**David Skinner**

South Africa

**David Smekal**

Sweden

**Sanju Sobnach**

South Africa

**Stephen Sollid**

Norway

**Kjetil Soreide**

Norway

**Eldar Soreide**

Norway

**Philip Stahel**

United Kingdom

**Giacomo Strapazzon**

Italy

**Manuel Florian Struck**

Germany

**Jose Suarez**

United States of America

**Geir Arne Sunde**

Norway

**Srinivas Susarla**

United States of America

**Gill Sviri**

Israel

**Hamid R. Talari**

Iran, Islamic Republic Of

**Julian Wei Tze Tang**

United Kingdom

**Colman Taylor**

Australia

**Brian Telesz**

United States of America

**Peter Teschendorf**

Germany

**Götz Thomalla**

Germany

**Tinne Tranberg**

Denmark

**Javier Urbano**

Spain

**Charles Wade**

United States of America

**Andrew Weatherall**

Australia

**Volker Wenzel**

Austria

**Jinliang Xing**

China

**Harlokesh Yadav**

India

**Junwei Yang**

China

**Hideo Yasunaga**

Japan

**Moriwaki Yoshihiro**

Japan

**Shi-Min Yuan**

China

**Erik Zakariassen**

Norway

